# A Bayesian Approach to Analyse Genetic Variation within RNA Viral Populations

**DOI:** 10.1371/journal.pcbi.1002027

**Published:** 2011-03-31

**Authors:** Trevelyan J. McKinley, Pablo R. Murcia, Julia R. Gog, Mariana Varela, James L. N. Wood

**Affiliations:** 1 Cambridge Infectious Diseases Consortium, Department of Veterinary Medicine, University of Cambridge, Cambridge, United Kingdom; 2 Department of Applied Mathematics and Theoretical Physics, University of Cambridge, Cambridge, United Kingdom; 3 Department of Veterinary Medicine, University of Cambridge, Cambridge, United Kingdom; University of California San Diego, United States of America

## Abstract

The development of modern and affordable sequencing technologies has allowed the study of viral populations to an unprecedented depth. This is of particular interest for the study of within-host RNA viral populations, where variation due to error-prone polymerases can lead to immune escape, antiviral resistance and adaptation to new host species. Methods to sequence RNA virus genomes include reverse transcription (RT) and polymerase chain reaction (PCR). RT-PCR is a molecular biology technique widely used to amplify DNA from an RNA template. The method itself relies on the *in vitro* synthesis of copy DNA from RNA followed by multiple cycles of DNA amplification. However, this method introduces artefactual errors that can act as confounding factors when the sequence data are analysed. Although there are a growing number of published studies exploring the intra- and inter-host evolutionary dynamics of RNA viruses, the complexity of the methods used to generate sequences makes it difficult to produce probabilistic statements about the likely sources of observed sequence variants. This complexity is further compounded as both the depth of sequencing and the length of the genome segment of interest increase. Here we develop a Bayesian method to characterise and differentiate between likely structures for the background viral population. This approach can then be used to identify nucleotide sites that show evidence of change in the within-host viral population structure, either over time or relative to a reference sequence (e.g. an inoculum or another source of infection), or both, without having to build complex evolutionary models. Identification of these sites can help to inform the design of more focussed experiments using molecular biology tools, such as site-directed mutagenesis, to assess the function of specific amino acids. We illustrate the method by applying to datasets from experimental transmission of equine influenza, and a pre-clinical vaccine trial for HIV-1.

## Author Summary

Characterising genetic variation in viral populations can have important implications in terms of understanding how viruses evolve within infected hosts. Modern sequencing technologies allow genetic information to be obtained faster, more affordably, and in much greater quantities than before. This allows new experimental procedures to be designed to explore aspects of pathogenesis that were previously unattainable, particularly with regard to mutations that occur at particular nucleotide sites that may confer a fitness advantage to the pathogen. This information can be used to study important issues such as the development of antiviral resistance, virulence, and/or changes in host-range specificity. Nonetheless, the experimental procedures used to generate the data can incorporate artefactual errors, and in order to optimise the information obtained from these studies techniques are required to characterise which sites exhibit mutations that may alter viral fitness. As both the depth of sequencing increases and the length of the region sequenced increases (e.g. moves to whole genomes rather than smaller segments), large numbers of sites will exhibit some form of variation, and hence development of a probabilistic method to define and extract these sites-of-interest becomes more important. We tackle this problem here using a Bayesian framework.

## Introduction

Reverse transcription-polymerase chain reaction (RT-PCR) is a common tool to generate copy DNA (cDNA) from RNA. All publicly available sequences of RNA viruses have been generated using this technique. The method consists of two steps: the first is an *in vitro* synthesis of cDNA from an RNA template in a reverse-transcription reaction (RT); and the second (PCR) consists of multiple cycles of DNA amplification using the cDNA generated in the RT step as a template. As in any other polymerisation reaction, misincorporations that result in artefactual mutations are generated during both steps, although at different rates (reverse-transcriptases lack proofreading activity and thus the RT step is more error-prone, while DNA polymerases exhibit various degrees of proofreading activity).

The current genomics revolution has generated thousands of sequences of complete RNA viral genomes. Sequences derived from the influenza viruses resource (http://www.ncbi.nlm.nih.gov/genomes/FLU/FLU.html) alone account for more than 175,000 as of October 2010. Indeed, the advent of novel and more affordable sequencing technologies allows the study of viral populations in an unprecedented depth, up to the level of characterising within-host viral populations in a qualitative and quantitative fashion. In particular, such studies are critical to understand the mechanisms that govern the evolution of virulence or antiviral resistance, as well as the underpinning mechanisms of cross-species jumps and immune evasion. In addition, in-depth studies of genetic variation are increasingly used to elucidate the viral population dynamics and evolution (phylodynamics) both within and between hosts [1].

Different laboratories have explored the within-host variation and evolution of a variety of RNA viruses, ranging from those that cause acute infections such as influenza and dengue [2]–[6], to those that persistently infect their host, like human and simian immunodeficiency viruses [7]–[10]. Despite differences in experimental design due to inherent biological features of the virus under study (i.e. specific host, inoculation route, replication strategy) and the biological questions being addressed (i.e. size of transmission bottlenecks, time of appearance of antiviral resistance or immune escape variants), most of these experiments rely on the analysis of sequences derived from viral samples taken at different times post-infection. The underlying assumption is that if multiple samples are taken from a single host over time, it is possible to map the frequency of a particular observed sequence and its variants in a temporal fashion. However, since there are various sources of error, both in the viral replication cycle and in the experimental process, it is difficult to elicit (probabilistically) whether observed variants are consistent with the possibility of viral evolution, or simply a result of random misincorporations occurring either within the host or during the RT-PCR/sequencing process. We propose a Bayesian method to try to make such distinctions, and to illustrate these techniques we use data from an experimental transmission study of equine influenza virus (EIV) in its natural host [6], and data from a prime-boost pre-clinical vaccine trial in a non-human primate model for HIV-1 (M. Varela and J. L. Heeney, *in preparation*).

**Figure 1:**
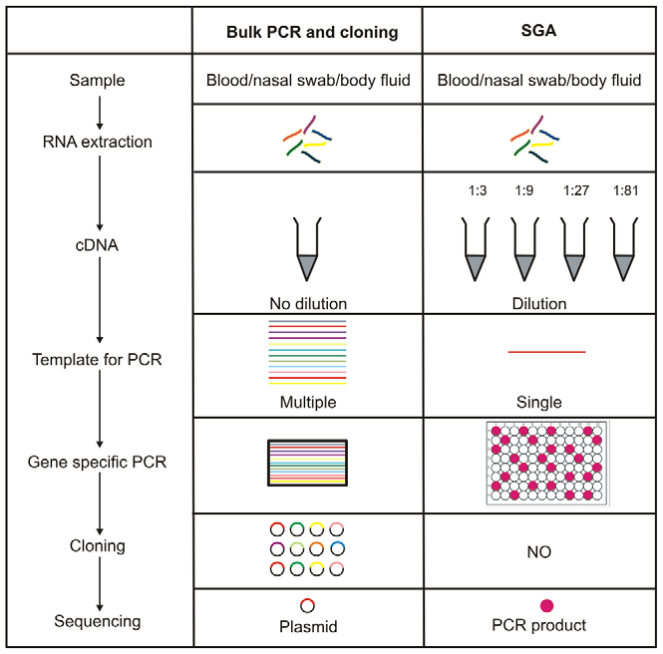
Schematic comparison of clonal vs. SGA sequencing.

An important biological distinction between these two pathogens is the duration of the infection; while influenza infections are typically acute, lasting for only a few days, HIV infections can last for a lifetime. In addition, the experimental procedures established for the study of within-host evolution for those two infections are different (Figure 1). For HIV, single genome amplification (SGA) followed by direct sequencing is currently the technique of choice [7], [8], [11]–[15]. In SGA, viral RNA is extracted from a clinical sample (typically a blood sample) and copied into cDNA, which in turn is subjected to a limiting dilution step such that only one molecule is then used as a template for a PCR reaction. Thus the obtained PCR products are the result of the amplification of one single molecule of cDNA. These PCR products are then sequenced directly without cloning. An alternative experimental approach is clonal sequencing, which has been used to study intra-host viral populations of influenza and dengue [2], [3], [5], [6]. With this method, RT-PCR is performed from a clinical sample, followed by subcloning of the resulting PCR products into sequencing vectors, which in turn are introduced into bacteria in order to produce the necessary quantities of DNA required for sequencing. In clonal sequencing, DNA from individual colonies (i.e. single molecules of PCR product) are extracted and sequenced.

The statistical framework we present here is quite general, and we show how it can be used for screening data from longitudinal within-host experiments, and/or between-host transmission studies. The mechanism by which we identify “sites-of-interest” is to monitor the frequency of bases present at a particular nucleotide site in the background population of viruses. It should be noted that the approach we propose here is not meant to replace methods to study selection analysis, for which there are already many excellent algorithms and software packages available (e.g. [16]). Instead the method is designed to flag up single sites that exhibit changes in the structure of the distributions of bases either over time, or relative to a reference sequence (such as that obtained from an inoculum sample). Furthermore it aims to provide a weight-of-evidence in favour of population structures that suggest higher frequencies of mutations than would be expected if all mutations arose randomly without further propagation (i.e. *de novo*). There are various biological mechanisms that could cause these observed changes, for example competition or selection within the host, and we discuss various options in more detail in the Materials and Methods and Discussion sections. The method can also be used to inform subsequent experiments that aim to target the role of individual nucleotide variants in defined phenotypes. In both studies described here, viral sequences have been generated using capillary sequencing technologies (i.e. Sanger sequencing). Although newer sequencing technologies that produce thousands of reads are available, they are not yet established for the kind of studies analysed here. This is due to the variable length of reads they produce (50 to 250 base pairs), which makes it difficult to link distant mutations, as well as for the intrinsic error rates they display.

## Materials and Methods

### Statistical methodology

The genetic units of interest here are individual nucleotide sites, and the output from the sequencing process is a distribution of bases present across a finite set of observed sequences. For consistency we define an observed ‘mutation’ to be a deviation away from the consensus base at a particular nucleotide site [14]. At a given nucleotide site the consensus is defined as the base present at the highest frequency in the set of observed sequences from the inoculum (for the HIV study) or the initial challenge animal (for the EIV study). In the event that there is no clear consensus base at a particular site (e.g. a 50∶50 split), then numerically the methods described subsequently are invariant to the choice of ‘consensus’ and ‘mutation’, though care must be taken with the biological interpretation of the results.

In the first instance we will consider an individual dataset containing *S* sequences of *N* nucleotides each, derived from a single clinical sample (in this case a blood sample or a nasal swab). At any single nucleotide site there are three possible deviations away from the consensus base. The distributions of observed bases at a single nucleotide site can then be considered as a random draw from the background population, and can be described by a multinomial distribution (described below).

More formally, if we denote the number of bases of type *B* at site *j* as 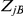, then the probability of observing 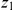 sequences with base 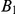, 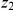 with base 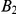, 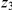 with base 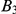 and 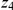 with the consensus base 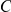 at position *j* is:
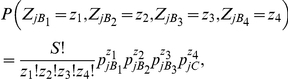
where 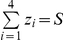 and 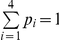. Here the parameters 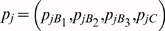 correspond to the proportion of each base present in the background population. For brevity we drop the complex subscript, such that 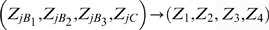 and 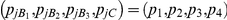; making only the concession that the consensus base is always indexed 4.

The goal of this work is to develop a screening mechanism to inform the development of future studies. The proposed method aims to identify nucleotide sites whose frequency of mutations differ from their expected values, which in turn are based on a given viral population and some simple assumptions about the mechanisms of random mutation events. We aim to approach this problem by using two main sources of information: the overall proportion of mutations present in the observed sequences (denoted 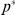), and multiple viral samples obtained over time (and/or within different animals). Given a starting population of viruses, consider initially the case that *all* observed mutations occur randomly *without* further replication. In this scenario the distribution of observed bases at a nucleotide site *j* will be expected to follow a multinomial distribution such that 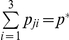, regardless of the background *structure* of the 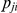
s.

On the other hand, if a site *j* exhibits a frequency of mutations such that 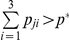, then it is much more likely that some form of amplification of one or more mutations has occurred, and these are defined as our “sites-of-interest”. Of course in reality 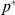 will contain both “unamplified” and “amplified” mutations, as it averages over all positions. Hence using the constraint 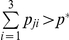 to characterise sites-of-interest will be conservative, in the sense that we are less likely to identify some truly amplified mutations due to the potential overestimate of 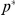. However, we are *not* modelling the biological mechanisms that cause the population structure 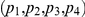 to change, and therefore it is necessary to consider the interpretation of sites identified using this criterion.

We note that any mutation must have occurred either by a biological mechanism (“real”), or as an artefact of the RT-PCR process (“artefactual”), and the aim of this work is to distinguish between these mechanisms in a viable manner. As in all practical discrimination algorithms there is the potential for classification error to happen, and in this case a false positive occurs when an *artefact* mutation is classified as a mutation-of-interest, and a false negative occurs when a *real* mutation is missed. In fact the distinction is more subtle than this, since real mutations that are either neutral or deleterious to the fitness of the virus are not usually of interest from a biological perspective, and if these occur then they are likely to be present at very low levels at any given time point and so will not be isolated via our screening criterion. Of course we also run the risk of missing real mutations that do confer a fitness advantage but have only just begun to replicate (i.e. they are present at low levels in the population). Our method cannot make the distinction between these “real” low frequency mutations and low frequency mutations occurring as a result of RT-PCR error (without a more complex mutation model). Instead we argue below that we if we can isolate high frequency mutations in a careful way, then these are more likely to constitute evidence of providing an increased fitness advantage to the virus, and hence are of particular biological importance.

Of course, it is possible that single-site mutations that *do* show evidence of replication could have arisen during the RT-PCR process. Although this is theoretically possible, we expect that this happens at such a negligible level that it is highly unlikely that mutations isolated during our screening mechanism would have arisen in this way. For example, in clonal sequencing we amplify a large population of viruses, and expect that the amplified population will show a similar structure to the original population. If anything we might expect to miss variants that are present at low levels, since there is some concern that clonal sequencing might bias towards picking up those variants present at high levels in the population [14], and hence we would be less likely to isolate mutations of this type using our screening criterion if this were true. In SGA the original populations are diluted down after reverse transcription in an attempt to amplify single viral molecules. In this case only mutations occurring in the RT step would count as artefacts. If an isolated mutation occurs in the early steps of the PCR and becomes amplified in the following cycles, such that it theoretically makes up a large enough proportion of the amplified population to be detected, then these sequences are removed from the analysis after visual inspection of the chromatograms. Thus errors at the PCR step are minimised.

Furthermore, if we sequence multiple clinical samples then the RT-PCR processes that generate the data will be independent for each of these samples. Therefore if we saw the same mutation occurring in *multiple* clinical samples it is even more unlikely that this has occurred as an artefact of the RT-PCR. In either case we acknowledge the possibility that an isolated mutation could be a false positive, but consider the probability to be negligible. We reiterate that the methods described here aim to screen the data for sites-of-interest, and there may well be a small degree of false positive mutations that creep in; however, an important point is that this false positive rate will be further mediated if we observe the same mutation in multiple clinical samples, either from the same or different hosts.

There is an additional subtlety however, and that is that the background population of viruses in the inoculum may not be homogeneous, and thus the variation in bases in a set of observed sequences may simply be a result of sampling from this heterogeneous background population. Therefore it is also of interest to compare the distributions of bases at a particular site to the distribution in the inoculum, or other earlier viral sample (e.g. animal source of infection in the EIV study). To this end we highlight the necessity to model both frequencies *and* distributions of mutations. If we were interested purely in the former, then we could produce the corresponding marginal binomial distribution modelling the *number* of mutations observed in a set of sequences. However, if viral evolution is or has occurred, it is possible that two viral populations will carry the same frequency of mutations, but of different types. Therefore we argue here that using a method based on the full multinomial model allows comparison of the distributions *and* frequency of observed mutations, rather than simply the latter.

To summarise, we have argued so far that we need to:

- screen for sites that show a higher frequency of mutations than expected if no propagation of these mutations had occurred, and in addition
- screen for sites that show changes in the distribution of bases compared to earlier viral samples.

These criteria then define a set of “sites-of-interest” that have a reasonable biological basis for exploration in future studies.

### Bayesian model choice

The question then arises as to how to derive a sensible method to elicit these sites. In a classical statistical framework we would generate a null hypothesis in each case and then ask the question: under this null hypothesis how likely are we to see an observation *at least as* or *more extreme* than the observed value? However, it is also only possible to build evidence against a *single* null hypothesis, and yet there are various random substitution models that may be appropriate [17]–[19], that would ascribe different structures to the background population of bases. For example, under the Jukes-Cantor substitution model [17] the frequencies of the four nucleotides at equilibrium would be 25%. In reality, a given nucleotide is much more likely to be miscopied as a *transition* than a *transversion* [19], and although this could be incorporated by setting different values for the proportions 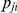 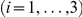 in our null model, these would have to be known beforehand or estimated from the data. Here we wish to compare between multiple competing models, and in addition we also want to compare between multiple distributions. The Bayesian method we propose presents a flexible alternative to both of these problems.

Also, often we do not know the specific site of interest in advance, and in a classical framework it would also be necessary to account for the number of nucleotide sites being studied. One way to do this would be to use a multiple correction procedure, such as the Bonferroni or Holm-Bonferroni corrections (that correct for the familywise error rate; see e.g. [20]), or the Benjamini-Hochberg correction (that controls for the false discovery rate; [21]). The choice of correction procedure depends on the context of the problem posed; the former are more stringent in protecting against false positives, whereas the latter allows a proportion of false positives to be obtained in order to increase the probability of detecting all true positives. In all cases the degree-of-correction depends on the number of independent tests (e.g. sites) evaluated.

The approach we propose here uses Bayesian models based on Bayes' Factors (BFs; [22]–[24]). In contrast to the classical statistical framework where the parameters of the system are assumed fixed, in a Bayesian framework all parameters are considered to be random variables with each following a probability distribution. As such it is possible to analyse competing models in an analogous way to that of a classical hypothesis test, but with various advantages, namely:

1. In a classical setting, hypothesis test are set-up to look for evidence *against* the null hypothesis; however, they do not provide weights-of-evidence *in favour* of the null hypothesis, nor in relation to competing alternative hypotheses. Both of these things can be done in a Bayesian framework.
2. If particular nucleotide sites are known in advance to be associated with the occurrence of non-deleterious or advantageous mutations, then it is possible to incorporate this information in the form of an increased *prior* probability of association.
3. This prior information can be used in an analogous way to multiple correction procedures, but is invariant to the number of tests performed, making it suitable for analysing very long sequences.
4. Useful probability measures, such as the posterior probability of association (PPA) can be produced to explore different associations, which are straightforward to interpret and can be combined to explore composite hypotheses. The PPA in this context represents the posterior probability that a nucleotide site exhibits the phenomena of interest (for example, high frequencies of mutations *and* differences between the distributions of bases obtained from the inoculum and a specific viral sample).

Other, more general advantages of BFs are described in Kass and Raftery [23], and an excellent introduction to the use of BFs in general, but specifically in genetic association studies can be found in Stephens and Balding [24].

Formally, the BF is defined as the posterior odds in favour of one model against another, when the prior probability of either model is equally favourable, and is defined as:
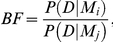
where 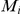 and 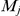 are competing models, and *D* is the observed data. We can view the competing models as competing hypotheses.

The Bayesian framework can be used to generate the PPA for a given model, and this can be generalised to multiple competing models. Let 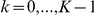 denote the competing models, and let 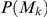 be the prior probability that model 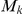 is correct, such that 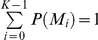. Then by Bayes' Theorem:
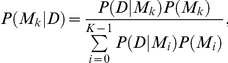
where
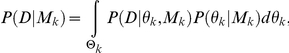
with 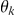 the (unknown) parameters on parameter space 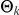. This approach therefore integrates, or averages (rather than maximises) over the parameter space.

If we are looking at multiple nucleotide sites, and 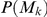 is equal across all sites, then 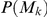 represents the prior *proportion* of sites that exhibit the phenomena of interest believed to exist in the population. This is similar to classical multiple testing procedures that account for the false discovery rate, but has the advantage that it does not depend on the number of tests performed, only the *proportion* of true associations believed to exist in the population [24].

### Generating comparative model structures

To attempt to identify sites-of-interest, we will specify a set of competing models that cover a range of feasible background population structures. Therefore the set of observed sequences corresponds to a random draw from one of these population structures. In many cases we have to resort to numerical methods to calculate the likelihood, 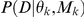, but for the models discussed here it is possible to derive these analytically (for mathematical details see Protocol S1). For brevity the subsequent discussion assumes that we are dealing with a single nucleotide site, and we drop the site subscript. The observed data at a site are denoted 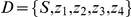, where *S* is the number of observed sequences.

For a set of sequences obtained from a single dataset (i.e. an individual clinical sample) we can define ten competing structures for the background population of bases at a given site. The first five models cover a range of population structures subject to the overall mutation rate being equal to 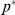, where 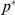 is the per-nucleotide mutation probability, i.e. the probability that a nucleotide in a randomly chosen sequence at a randomly chosen site differs from the consensus. We estimate 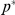 by computing the overall proportion of mutations present in the data.
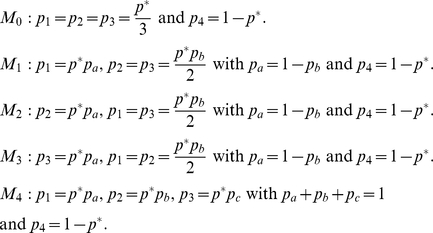
Furthermore, we can also specify an analogous range of models in which the overall mutation rate *p* is allowed to vary between 0 and 1.
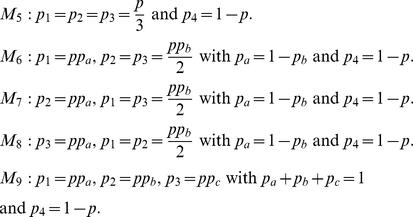
The derivation of 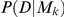 for each of these models is discussed in Protocol S1 and mathematical forms given in Table S1, along with R [25] functions to evaluate these probabilities.

### Extension to multiple viral samples

If multiple viral samples are available (i.e. clinical samples obtained at different times post-infection), 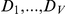, then it is necessary to introduce some additional notation to capture the fact that different samples could have arisen as a result of sampling from different background populations. For example, consider that data from two viral samples from the same animal are available, denoted 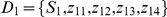 and 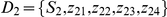. There are two possible scenarios: either *D*_1_ and *D*_2_ are random samples from the *same* population, or they are random samples from *different* populations. We make the assumption that at any time the population of bases at a given nucleotide site will be consistent with one of the models 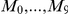, and we denote the combination of models that could explain the data by using multiple subscripts corresponding to the viral sample i.e. 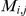, where 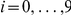 corresponds to the population structure for the first viral sample (*D*_1_) and 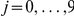 to the structure for the second viral sample (*D*_2_).

**Figure 2:**
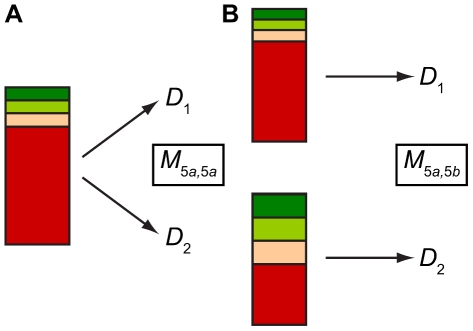
Schematic diagram of sampling from the same or different populations exhibiting the same population structure (here based on 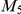
 **).**

Thus it is necessary to calculate 
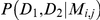
 for any *i* and *j*. If 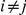, then by definition the background populations from which *D*_1_ and *D*_2_ are sampled are different, and so 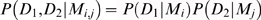
—see Protocol S1. When 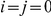 then there are no free parameters over which to integrate, and so 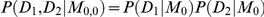. If 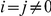 then there is an additional subtlety, in that *D*_1_ and *D*_2_ are *either* sampled from the *same* population, or from two different populations but with the same structure. To try to clarify this point, consider Figure 2. This shows the case when 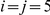. In Figure 2A we see that *D*_1_ and *D*_2_ are random samples from the *same* population described by the model 
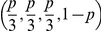
. In Figure 2B we can see that *D*_1_ and *D*_2_ are random samples from two different populations, but with the same population structure, described by 
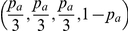
 and 
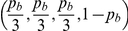
 respectively.

To differentiate between these possibilities we introduce an additional character subscript such that cases similar to Figure 2A are denoted 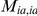 and cases similar to Figure 2B as 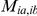. The main difference in the calculations of 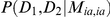 and 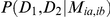 relate to the parameter space over which it is necessary to integrate. These results follow from the fact that although the background populations are not independent, the sampling mechanism is. Mathematical detail is given in Protocol S1.

Summarising for the two-sample case, we have 
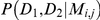
 given by:
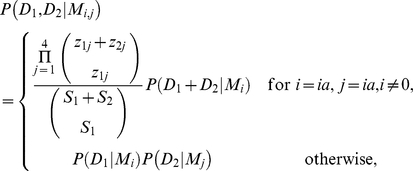
where 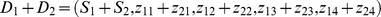. Hence there are 109 possible competing models that could explain the joint distribution of *D*_1_ and *D*_2_, since there are 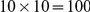 possible ways of producing random samples from two *different* background populations based on structures 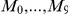, and a further 9 ways corresponding to when *D*_1_ and *D*_2_ are samples from the *same* background population (based on 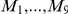). It is possible to generalise these calculations to more than two viral samples as required, and hence it is possible to produce a PPA for all possible combinations of potential background structures.

### Screening for sites-of-interest

Given sequence data from multiple viral samples, we have described a method that produces weights-of-evidence in favour of the data being drawn from a particular configuration of background populations. In effect these population structures can be used to define various criteria-of-interest, which can then be assigned an overall PPA by summing across the relevant models. This can then be used to provide useful information about potential changes in the background population of viruses (if any), and whether or not the frequency of mutations is higher than we would expect if there had been no propagation of mutations (so all mutations are first generation). In this case we can combine the two types of sites we are seeking into a single question that can be tested using this framework:

“What is the probability that *at least one* clinical sample exhibits a higher frequency of mutations than expected if no propagation of these mutations has occurred, and also shows a different background structure to the inoculum?”

To calculate this we can append the inoculum to the observed data and treat it as an additional sample. We can then sum over the corresponding model structures that are consistent with the question of interest. In the two sample case, we have data 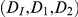, where 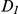 is the data for the inoculum (or initial challenge animal) and we denote the PPA for this definition of site-of-interest as 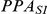, which can be calculated as:
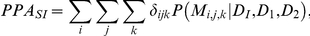
where
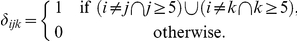
It is worth noting here that a range of questions could be asked of the data, for example we may be more stringent and ask for the probability that *all* viral samples obtained from an animal show a different background structure to the inoculum and exhibit a higher frequency of mutations than expected if no propagation has occurred. In which case,
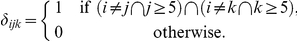
At the current time we use a brute-force computational approach to calculate the PPAs for all models, however it would be possible to develop an approximation based on a variation of the Occam's Window approach of [26] in order to make the calculations less computationally intensive for particularly large-scale problems.

### Data and study designs

#### 1. A model of natural transmission of EIV in horses

**Figure 3:**
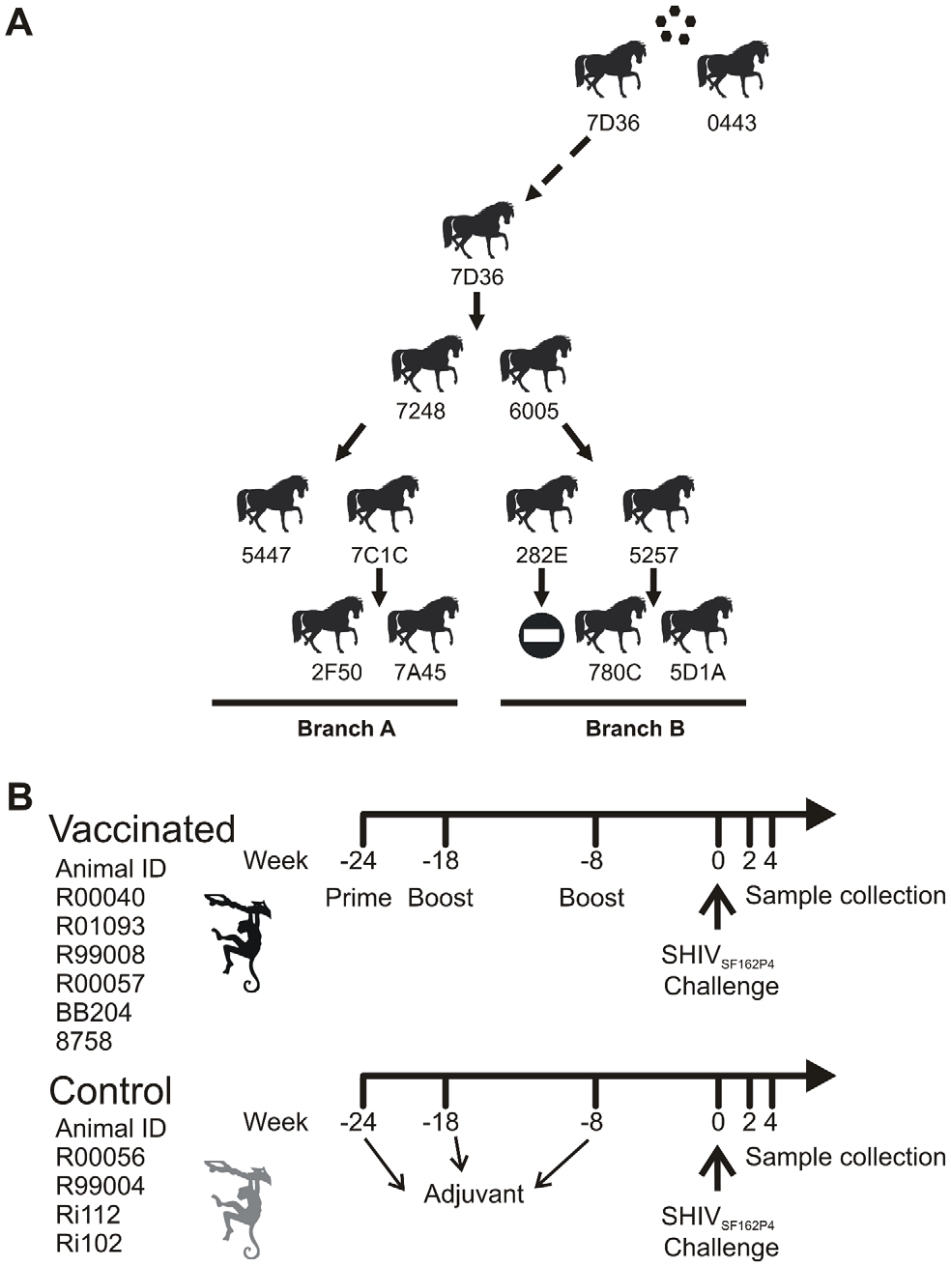
Experimental designs for A: EIV, and B: HIV-1 studies. Animal clipart reproduced from www.openclipart.org under the CC0 1.0 Universal Public Domain Dedication license.

A transmission chain was established by experimentally infecting two horses and housing one of them with two naïve horses in the same stable until the “recipients” showed clinical signs of infection. At that point, the recipient horses were separated and each was housed together with another pair of horses (Figure 3A). Nasal swabs were taken from infected animals on a daily basis and viral RNA was extracted for RT-PCR amplification, subcloning and further sequencing of individual clones to determine the mutational spectra of within-host viral populations (for a detailed account see [6]).

Multiple sequences from each daily sample were generated by capillary sequencing and compared to the sequence of the seeder horse. A key aim was to identify single-site mutations arising in viral samples that were unlikely to simply be artefacts of the experimental process. It was of particular interest to detect variants that persisted for multiple days within a host, or were transmitted between horses.

#### 2. Non-human primate pre-clinical vaccine model for HIV-1

The aim of this study was to identify specific changes occurring in the HIV-1 envelope glycoprotein within a host under selective immune pressure elicited by neutralizing antibodies. To this end, a prime-boost pre-clinical vaccine trial was performed in rhesus macaques. Six animals were subjected to a prime-boost vaccine regime comprising a combination of recombinant gp140 envelopes from clades A, B and C and envelope peptides while four animals were used as controls (M. Varela and J. L. Heeney, unpublished results; see Figure 3B). Plasma samples were collected two and four weeks after challenge with HIV-1_SF162 P4_ virus stock. Viral RNA was extracted and envelope genes were PCR amplified using single genome amplification (SGA) followed by direct sequencing as previously described [14].

## Results

For consistency in this section, we report all the results to two significant figures (s.f.). Sequences containing insertions were removed from the data sets, and deletions at particular sites discounted the number of bases entered into the analysis for that site.

### EIV study

The data in [6] consist of 2366 sequences of length 903 nucleotides, derived from 30 samples taken from 11 horses over a 15-day experiment. The number of sequences derived per sample ranged from between 44 and 154 sequences, and the breakdown by individual sample is shown in Table 1.

**Table 1:** Number of sequences obtained by animal and date from EIV study [6].

Horse	Day	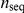
**7D36** *	3	152
	5	82
**7248**	5	154
	6	65
	7	154
	8	62
**6005**	5	67
	6	52
	7	72
	8	81
**5447**	8	69
	9	44
	10	50
	11	51
**7C1C**	7	83
	8	75
	9	52
	11	54
**5257**	7	112
	8	127
**2F50**	11	49
	12	54
	13	80
	15	46
**7A45**	11	107
**780C**	11	71
**5D1A**	12	54
	13	81
	14	79

*indicates the first naturally challenged animal.

The aim of this study was to determine the levels of genetic variation along the course of infection in single horses and how transmission can impact on the process of within-host evolution. As such we can screen the sequence data to identify nucleotide sites that have specific properties of interest as defined earlier. The methods described here could help to locate sites and mutations that confer some fitness advantage, or perhaps neutral selection through drift founder effects which can also give some insight into the viral population dynamics.

To calculate the required PPAs it is necessary to specify a *prior* probability of association for each of the competing models. We suggest using a range of priors to assess the strength of any observed associations. Here we choose values of 0.001, 0.01 and 0.05 in favour of the phenomena of interest (as defined in the Materials and Methods), split uniformly across all model structures consistent with being sites-of-interest. The remainder is split uniformly across all model structures inconsistent with our definition. Table 3 provides the PPAs that *at least one* sample exhibits differences in the background population structure compared to the initial challenge horse, that also shows a higher frequency of mutations than we would expect if no replication occurs (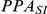). It can be seen that even with a very low prior probabilities (0.001; so less than one site *a priori*) there are three sites that show very strong evidence of an association (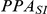
>0.97), and three that show some evidence (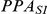
>0.07).

What this method allows us to do is to observe *how* the distributions are changing between samples, which provides useful information on which sites/mutations may be changing over the course of the experiment. It is therefore possible to explore specific sites in particular horses in more detail. As an illustrative example consider site 478 in horse 5447. This had 4 samples taken on days 8–11 (after initiation of the transmission chain), generating 69, 44, 50 and 51 sequences respectively (Table 1). It can be seen that the only time point at which mutations are observed is on day 11 (Table 4). To model this it is necessary to treat the samples from the challenge horse as an additional sample (also shown in Table 1), which results in 210 979 possible sets of models that could explain the output from the five viral samples. Table 5 shows the first five models returned after sorting the output by decreasing PPAs. Notice that the PPAs in favour of each of these models is equal, and this is because in the situation where there are no observed mutants in a set of sequences, then there is not enough information in the data set to distinguish between 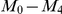 (see Table S1). What is driving this pattern is the fact that on day 11 the technique is selecting 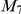 to be the most likely background population to have given rise to the data. Hence model 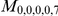 returns the same PPA as model 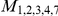, or any other model that uses structures 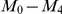 for the samples from the challenge horse and days 8–10. It is the sum over all models that have structure 7 on day 11 that is potentially of more interest here, which results in a marginal probability of 0.54.

We can repeat this for all possible models for day 11, with 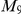 having a marginal probability of 0.37 and 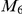 of 0.05. We have already discussed the likely biological mechanisms behind these mutations in the Materials and Methods, and these results provide strong evidence that the mutations observed in position 478 are likely to indicate real variation and replication within this host between days 10–11, when mutations of both type G478A and G478T occur. Interestingly, both G478A and G478T constitute non-synonymous mutations in a putative antigenic site.

### HIV study

The data in the HIV-1 study (M. Varela and J. L. Heeney unpublished) consist of 439 envelope sequences of length 2544 nucleotides, derived from 10 individuals (plus the inoculum) at two time points over a four week period (Table 2). The purpose of the study was to identify specific changes in the HIV-1 envelope glycoprotein within a host under selective immune pressure elicited by a prime-boost vaccine. The same questions as for the EIV study can be asked, though in this case the background population of viruses in the inoculum is much more heterogeneous (data not shown) than that from the initial challenge horse in the EIV study. Table 6 shows the results from those sites with 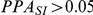 (with a 0.001 prior). Interestingly, and importantly, some of these sites are identified in more than one animal, even though this was not a transmission study. It is also possible to split the results by vaccination status. Qualitatively at least, the results in Table 6 suggest that more sites show deviations from the inoculum in the vaccinated group than the unvaccinated group, which is suggestive perhaps of increased diversification due to selection pressure in response to the vaccine.

**Table 2:** Number of sequences obtained by animal and date from HIV-1 study (M. Varela and J. L. Heeney, *in preparation*).

Animal	Week	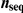
**Stock** *		22
**8758**	2	40
	4	17
**BB204**	2	22
	4	8
**R00040**	2	27
	4	6
**R00056**	2	29
	4	19
**R00057**	2	42
	4	10
**R01093**	2	24
	4	35
**R99004**	2	19
	4	41
**R99008**	2	9
	4	10
**Ri102**	2	15
	4	17
**Ri112**	2	12
	4	20

*indicates inoculum sequence.

**Table 3:** Posterior probability of association, 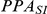, for different sites from the EIV study.

Horse	Position	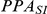 (0.001)	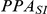 (0.01)	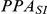 (0.05)
5447	478	1.0	1.0	1.0
6005	49	1.0	1.0	1.0
6005	884	0.98	1.0	1.0
2F50	231	0.18	0.69	0.92
7248	134	0.09	0.50	0.84
6005	61	0.08	0.47	0.82

Parentheses show the *prior* probability of association across all models of interest at a site. Sites shown are those in which the 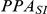 for the smallest prior (0.001) is >0.05.

**Table 4:** Frequency of bases for site 478 in horse 5447 in the EIV study.

Base	Challenge horse	Day 8	Day 9	Day 10	Day 11
**G** *	152	69	44	50	37
**A**	0	0	0	0	8
**C**	0	0	0	0	0
**T**	0	0	0	0	6

*Consensus base.

**Table 5:** Summary of models and PPAs for site 478 in horse 5447 from the EIV study.

Model	PPA
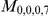	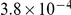
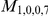	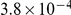
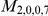	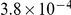
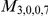	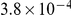
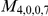	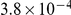
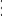	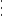

**Table 6:** Posterior probability of association, 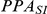, for different sites from the HIV-1 study.

	Unvaccinated			Vaccinated		
Position	(0.001)	(0.01)	(0.05)	(0.001)	(0.01)	(0.05)
1518				1.0	1.0	1.0
1518				0.27	0.79	0.95
1449	1.0	1.0	1.0			
491	1.0	1.0	1.0			
2387				1.0	1.0	1.0
994				1.0	1.0	1.0
994				0.99	1.0	1.0
994	0.15	0.64	0.90			
1006				1.0	1.0	1.0
1006				0.99	1.0	1.0
1006	0.15	0.64	0.90			
1285	1.0	1.0	1.0			
1744				1.0	1.0	1.0
1752				1.0	1.0	1.0
1752				1.0	1.0	1.0
1752	1.0	1.0	1.0			
2470				1.0	1.0	1.0
2470	1.0	1.0	1.0			
449				1.0	1.0	1.0
836	1.0	1.0	1.0			
2219				1.0	1.0	1.0
393	1.0	1.0	1.0			
393				0.37	0.86	0.97
756				1.0	1.0	1.0
756	0.06	0.39	0.77			
433				0.99	1.0	1.0
433				0.14	0.62	0.90
771				0.99	1.0	1.0
771				0.07	0.44	0.80
942				0.99	1.0	1.0
273				0.99	1.0	1.0
2290				0.97	1.0	1.0
2446				0.97	1.0	1.0
750				0.94	0.99	1.0
138	0.89	0.99	1.0			
138				0.05	0.35	0.74
1644				0.79	0.97	1.0
418				0.77	0.97	0.99
7				0.74	0.97	0.99
406	0.70	0.96	0.99			
406				0.15	0.65	0.90
1305				0.60	0.94	0.99
1305				0.56	0.93	0.99
504				0.39	0.86	0.97
1512				0.39	0.86	0.97
1792	0.31	0.82	0.96			
1525				0.31	0.82	0.96
2492				0.26	0.78	0.95
680				0.25	0.77	0.95
1347				0.25	0.77	0.95
1479	0.15	0.64	0.90			
2007				0.13	0.61	0.89
777				0.11	0.56	0.87
270	0.11	0.54	0.86			
386				0.09	0.51	0.85
1668				0.09	0.51	0.85
1134				0.09	0.50	0.84
475	0.09	0.50	0.84			
2340	0.09	0.50	0.84			
426				0.08	0.48	0.83
445				0.08	0.48	0.83
46				0.06	0.39	0.77

Each line corresponds to a different animal. Values in parentheses show the *prior* probability of association across all models of interest at a site and reported sites are those for which the 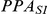 with the smallest prior (0.001) is >0.05.

It is possible as before to delve further into the nature of the mutations observed, and how the distributions have changed. For example, consider site 994; this site is identified in three animals (R01093, BB204 and R99004), two vaccinated and one unvaccinated (though the PPA is weaker for the unvaccinated animal). A summary of bases for each animal at each time point are shown in Table 7. It is clear to see that there has been a change of consensus over the time course of infection in each of these animals, switching from G in the inoculum sample to A in each of the subsequent samples. This is backed up further by the PPAs for different model structures (Table 8), in which model 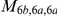 was selected as the most likely model structure in two cases (PPAs of 0.78 and 0.73 respectively) and as the second most likely model structure in the third (PPA = 0.12). In the latter case the most likely model was 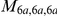 (PPA = 0.82), which suggests that structure 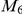 was the most likely for each sample, but that given the sample size it was not able to fully disregard the possibility of random sampling from the inoculum (note that in the HIV-1 study there were less sequences produced per sample, and hence an increase in variability in the accuracy of the estimated distributions – nevertheless more sites-of-interest were identified overall). Of key importance is the fact that this site was picked up in multiple animals, and so for reasons discussed previously these differences are highly unlikely to have arisen as a result of independent RT-PCR error.

**Table 7:** Frequency of bases for site 994 in animals R01093, BB204 and R99004 in the HIV-1 study.

	Inoculum	R01093		BB204		R99004	
		Wk 2	Wk 4	Wk 2	Wk 4	Wk 2	Wk 4
**G** *	15	0	0	0	0	1	8
**A**	7	24	33	20	8	13	31
**C**	0	0	0	0	0	0	0
**T**	0	0	0	0	0	0	0

*Consensus base.

**Table 8:** Summary of models and PPAs for site 994 in animals R01093, BB204 and R99004 in the HIV-1 study.

R01093		BB204		R99004	
Model	PPA	Model	PPA	PPA	PPA
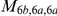	0.78	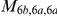	0.73	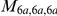	0.82
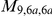	0.17	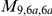	0.16	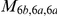	0.12
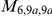	0.03	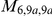	0.05	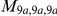	0.03
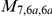	0.01	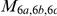	0.02	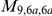	0.02
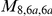	0.01	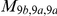	0.02	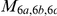	0.01
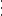	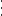	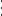	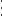	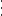	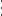

In the EIV study we did not observe any sites that showed mutations occurring at a high frequency in more than one sample, however in the HIV-1 study there are various occurrences of this nature (such as at site 994). It is possible to screen specifically for these mutations specifically by placing more stringent criteria on the data; namely that we wish to identify mutations in which the data show evidence of deviating from the inoculum in *both* samples, as well as showing a high frequency of mutations from the consensus. These are shown in Table 9. Although the absolute probabilities are different (due to the resulting change in prior caused by the change in the number of models-of-interest), the sites observed in Table 9 are all a subset of those identified in Table 6 (with the exception of site 261 in animal 8758, which has a low PPA in any case). This illustrates a practical way in which these methods can be adapted to deal with specific questions.

**Table 9:** Posterior probability of association, 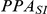, for different sites using a more stringent criterion from the HIV-1 study.

	Unvaccinated			Vaccinated		
Position	(0.001)	(0.01)	(0.05)	(0.001)	(0.01)	(0.05)
1449	1.0	1.0	1.0			
1518				1.0	1.0	1.0
1518				0.66	0.95	0.99
491	1.0	1.0	1.0			
994				1.0	1.0	1.0
994				0.82	0.98	1.0
994	0.43	0.89	0.98			
1006				1.0	1.0	1.0
1006				0.82	0.98	1.0
1006	0.43	0.89	0.98			
1752				1.0	1.0	1.0
1752				0.97	1.0	1.0
1752	0.90	0.99	1.0			
2470				1.0	1.0	1.0
2470	1.0	1.0	1.0			
1285	1.0	1.0	1.0			
836	1.0	1.0	1.0			
756				1.0	1.0	1.0
756	0.23	0.75	0.94			
393	0.99	1.0	1.0			
393				0.61	0.94	0.99
2290				0.98	1.0	1.0
2446				0.98	1.0	1.0
449				0.94	0.99	1.0
138	0.85	0.98	1.0			
1305				0.79	0.97	1.0
1305				0.36	0.85	0.97
2219				0.75	0.97	0.99
504				0.46	0.89	0.98
1512				0.46	0.89	0.98
771				0.39	0.87	0.97
1134				0.32	0.82	0.96
406				0.28	0.80	0.95
750				0.27	0.79	0.95
750	0.09	0.51	0.84			
1479	0.15	0.65	0.90			
261				0.10	0.54	0.86

Each line corresponds to a different animal. Values in parentheses shown prior probabilities of association and reported sites are those for which the 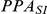 with the smallest prior (0.001) is >0.05.

## Discussion

Obtaining viral genetic information at multiple times post-infection either along the course of infection or along a chain of transmission, whether experimental or observational, can help us to understand the underpinning mechanisms that shape viral evolution. Nonetheless, it is difficult to obtain probabilistic information about whether these observed mutations are consistent or inconsistent with having occurred due to random mutation error. This information can provide insight into the potential fitness of single-site mutations, both in terms of survival within a host and transmission between hosts. To this end we have discussed various ways in which probabilistic measures can be derived in order to address specific questions regarding the pattern of observed single-site mutations in the data, and have applied these measures to two datasets derived from experimental studies on HIV and influenza. It should be noted that the approach we propose here is not meant to replace methods to study selection analysis but to complement them. Indeed, in [6] (for the EIV study) we estimated the mean numbers of non-synonymous substitutions per site and synonymous substitutions per site using the SLAC algorithm available in Datamonkey [16]. Interestingly, the mutations that we have picked in this manuscript as sites-of-interest were not identified by the aforementioned selection analysis. As a result of these more detailed analyses, we are now more confident than before in the findings of [6], that 4 of the 11 mutations present in individual horses on multiple days were real (sites 49, 61, 231 and 884; the other 7 identified in [6] were present in multiple samples but at low frequencies). In addition we picked up a further two mutations using our screening criteria, at sites 134 and 478. The latter was picked up at one time point in multiple horses in [6], and the former occurred at one time point in one horse, and so wasn't explicitly reported in [6]. However, it occurred with a high enough frequency of mutations to be detected here.

The intention of this work is twofold: first, to screen large data sets for mutations of interest, and second, to focus in more detail on highlighted mutations to elicit information about the change in background population structure across multiple samples. Whilst it is possible to generate classical hypothesis tests to tackle certain questions, we provide a method based on Bayesian model selection, for various reasons. The first is that it is possible to generate evidence *in favour* of a particular hypothesis, rather than simply weights of evidence against the null hypothesis. Also, it is possible to compare *multiple* competing hypotheses in a straightforward manner. The Bayesian framework also allows the inclusion of prior information regarding the probability of specific individual nucleotide sites to be linked to the occurrence of non-deleterious or advantageous mutations. When these prior probabilities take the same values for all sites, then they represent the prior proportion of sites thought to be associated in some way, which is similar in principal to the false discovery rate used in classical multiple correction procedures but is invariant to the number of sites being examined. This makes it particularly suitable for analysing long sequences (i.e. those ones generated by capillary sequencing). In many situations this prior information may not be available, and so it is necessary to conduct some form of sensitivity analysis to examine the strength of the posterior association for a range of prior values. This step helps to shed additional light on the robustness of the conclusions in the absence of detailed prior information. Moreover, in this Bayesian approach we integrate over the range of the unknown parameters, which means that the *structure* of the background population has to be specified, but the proportions do not have to be directly estimated (as would be necessary in a maximum likelihood framework). This allows for alternative hypotheses to be generated that assume that multiple samples can come from either the same, or different background populations or population structures.

The Bayesian method produces a posterior probability that a particular hypothesis is true, and can be extended to deal with sequences derived from multiple samples. This means that once a suitable range of competing model structures has been developed, different probabilistic questions can be asked of the data. For example, when analysing the EIV data we originally screened for sites that showed evidence of the phenomena-of-interest in *at least one* of the samples obtained from one animal. In contrast, in the case of the HIV-1 data, it was possible to apply more stringent criteria, which screened for sites that showed evidence of the phenomena-of-interest in *all* the samples. An important point is that the question asked will depend highly on the biological context of the problem, but the methodology is flexible enough to allow many probabilistic questions to be posed. It is worth adding at this point that the same framework could be used to screen for other types of change. For example, in the HIV-1 study the population of viruses in the inoculum was highly heterogeneous, and it would be perfectly possible to screen for initially heterogeneous sites that revert to a homogeneous population over time. The only difference would be a change in the definition of “sites-of-interest”. In addition it is worth noting that although the data analysed here have been obtained through experimental studies, this is not necessary for the methodology to be applied, though it may alter the interpretation of the results. It would be perfectly possible to apply the same techniques to observational data as might be obtained in a real-life disease outbreak.

What this method does not model explicitly are the underlying mechanisms behind observed systematic mutations. If the amplification and sequencing steps are faultless and therefore introduce no errors, then the identified mutations must exist or occur as part of the replication process in the background viral population. The techniques described here cannot make the distinction between low frequency mutations that may have occurred through viral replication or artefactual error, however they can help to distinguish between likely deleterious mutations or non-advantageous mutations and those that show signs of persistence. It also allows us to compare the distributions of bases at a given site with other populations, such as the inoculum. Furthermore, mutations that occur in more than one animal can happen either *de novo* within each animal or due to transmission, and one area of future development would be in extending these methods to include information regarding mutations observed in multiple *animals* explicitly into the PPA calculations.

As previously mentioned, different techniques (i.e. clonal sequencing and SGA) are commonly used for the study of HIV and influenza within-host evolution. Although it is beyond the scope of this study to argue the relative merits of the two techniques, it has been argued that SGA provides a more realistic representation of the viral populations under study as it avoids the generation of recombinant sequences due to template-switching and facilitates the detection of *Taq* polymerase errors [14]. However, this seems to be more important for studies of HIV than influenza, and since it is time consuming and expensive other methodologies are normally used to study intra-host evolution of other viruses. Nonetheless, as highlighted in the introduction, the experimental process to generate viral sequences is not fully efficient and so there is a non-zero probability of introducing artefactual errors. Figure 1 provides a simple schematic diagram comparing SGA to clonal sequencing, and highlights areas where errors could be introduced.

Recently there have been some methodological developments in estimating true mutation rates that account for bias and selection [27], and it would be possible to change the value of the overall mutation probability 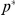 to accommodate this. It is worth noting that in terms of screening for true changes in the distribution of bases at particular sites as defined here, the values of 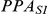 obtained for the within-sample problem will be *conservative* (i.e. will have a higher false negative rate), since the observed per-nucleotide mutation probability will include both artefact *and* real mutations.

It is also possible to conduct various control experiments to quantify the amount of error that occurs during various steps of the process. The experimental procedure used to generate the sequences in [6] included four sequential steps of DNA synthesis (generation of cDNA, PCR, DNA replication in bacteria and capillary sequencing). The main issue is determining the level of artefact mutations introduced during the reverse transcription, as this is likely to be the principal source of such errors. An issue is that these errors cannot be easily directly estimated experimentally as this will require the synthesis of a template RNA population made of identical RNA molecules, and there is no *in vitro* transcription system available that uses enzymes with proofreading activity. Moreover, the level of RT errors may vary with different template sequences, intracellular environment, and species origin of the RT enzyme. As a result it is difficult to draw firm conclusions as to the levels and sources of non-systematic error within sequences derived from a single sample without being able to directly quantify this error. Hence mutants that appear multiple times may either arise due to mutation events *de novo* in each sample, result from transmission from another animal, or be due to systematic errors in the RT-PCR steps (e.g. if particular sites/mutations are amplified in a highly non-uniform manner). However, as we discuss in detail in the Materials and Methods, there are various reasons that we do not think that we are likely to pick up changes in the distributions that are purely artefacts of RT-PCR errors using the screening criteria we introduce here. The probability of a result being a false positive is further diminished if a more stringent criteria is used (requiring evidence across multiple samples), or if similar changes are observed in multiple animals.

There is also the issue of sampling bias, however there is no reason to assume that systematic bias should creep into either the swab sampling or in the RNA extraction. Since, by producing a set of sequences, we are effectively taking a small sample from a large population, then the effect of sampling bias (if any) is most likely to be that rare mutations will constitute a very low probability of detection and a high probability of being missed during sampling. Therefore if we do identify sites-of-interest using the criteria defined here, then it is even more likely that these mutations would have to be present in reasonably high levels in the background population to be detected in this manner. This is reflected also in the increase in variability observed when smaller numbers of sequences are analysed.

Flexible probabilistic methods such as proposed here can help to elicit patterns from these complex and large-scale data sets based on asking intuitive questions about the data. We have described a method that allows improved inference from studies of viral transmission and evolution, in particular regarding the probabilities of observing particular mutations in viral sequence data. These types of study are becoming more common with the advent of deep and affordable sequencing technologies. Although the techniques presented here are based on data generated from capillary sequencing, they form a strong basis for developing algorithms specifically aimed at data generated by next generation sequencing technology. For example, sequences obtained using the Illumina platform can display substantial heterogeneity with regard to the depth of coverage across the genome segments after alignment. This means that more information (e.g. samples) will be available at some sites than others. This heterogeneity in information is intrinsically incorporated into the PPAs for individual sites through the Bayesian model specification. However, it will also be necessary to incorporate additional sources of error intrinsic to the specific platform being used, and this is the focus of ongoing work.

## Supporting Information

Table S1

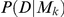
 for different population structures, along with associated R code for calculating 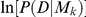.(PDF)

Protocol S1 Derivations of marginal distributions of the data and a protocol for dealing with multiple viral samples.
(PDF)
