# Supplementary material for: A Bayesian Approach to Analyse Genetic Variation within RNA Viral Populations
Source: PLoS Comput Biol. 2011 Mar 31;7(3):e1002027. doi: 10.1371/journal.pcbi.1002027 (PMC3068928; doi:10.1371/journal.pcbi.1002027)
Supplement: Table S1 — for different population structures, along with associated R code for calculating . (PDF) [file pcbi.1002027.s001.pdf]

Table S1: $P(D|M_k)$ for different population structures, along with associated R code for calculating $\ln[P(D|M_k)]$

In each case:

- `S` is the number of sequences (`=sum(z)`),
- `z` is a vector of counts, ordered such that `z[4]` is always the consensus base, and
- `pstar` is the estimated overall mutation probability p^*.

**Model**	$P(D\|M_k)$	**R code on log-scale**
$M_0 : p_1 = p_2 = p_3 = \frac{p^*}{3}$ and $p_4 = 1 - p^*$ with p^* known	$\frac{S!}{z_1!z_2!z_3!z_4!}\left(\frac{p^*}{3}\right)^{S-z_4}(1-p^*)^{z_4}$	`lfactorial(S)-sum(lfactorial(z))+ (S-z[4])*(log(pstar)-log(3))+z[4]*log(1-pstar)`
$M_1 : p_1 = p^* p_a, p_2 = p_3 = \frac{p^*(1-p_a)}{2}$ and $p_4 = 1 - p^*$	$\left(\frac{1}{2}\right)^{z_2+z_3}\frac{S!(z_2+z_3)!}{z_2!z_3!z_4!(S-z_4+1)!}(p^*)^{S-z_4}(1-p^*)^{z_4}$	`-(z[2]+z[3])*log(2)+lfactorial(S)+ lfactorial(z[2]+z[3])-sum(lfactorial(z[-1]))- lfactorial(S-z[4]+1)+(S-z[4])*log(pstar)+ z[4]*log(1-pstar)`
$M_2 : p_2 = p^* p_a, p_1 = p_3 = \frac{p^*(1-p_a)}{2}$ and $p_4 = 1 - p^*$	$\left(\frac{1}{2}\right)^{z_1+z_3}\frac{S!(z_1+z_3)!}{z_1!z_3!z_4!(S-z_4+1)!}(p^*)^{S-z_4}(1-p^*)^{z_4}$	`-(z[1]+z[3])*log(2)+lfactorial(S)+ lfactorial(z[1]+z[3])-sum(lfactorial(z[-2]))- lfactorial(S-z[4]+1)+(S-z[4])*log(pstar)+ z[4]*log(1-pstar)`
$M_3 : p_3 = p^* p_a, p_1 = p_2 = \frac{p^*(1-p_a)}{2}$ and $p_4 = 1 - p^*$	$\left(\frac{1}{2}\right)^{z_1+z_2}\frac{S!(z_1+z_2)!}{z_1!z_2!z_4!(S-z_4+1)!}(p^*)^{S-z_4}(1-p^*)^{z_4}$	`-(z[1]+z[2])*log(2)+lfactorial(S)+ lfactorial(z[1]+z[2])-sum(lfactorial(z[-3]))- lfactorial(S-z[4]+1)+(S-z[4])*log(pstar)+ z[4]*log(1-pstar)`
$M_4 : p_1 = p^* p_a, p_2 = p^* p_b, p_3 = p^*(1-p_a-p_b)$ and $p_4 = 1 - p^*$	$2\frac{S!}{z_4!(S-z_4+2)!}(p^*)^{S-z_4}(1-p^*)^{z_4}$	`log(2)+lfactorial(S)-lfactorial(z[4])- lfactorial(S-z[4]+2)+(S-z[4])*log(pstar)+ z[4]*log(1-pstar)`

**Model**	$\boldsymbol{P\left(D \mid M_k\right)}$	**R code on log-scale**
$M_5: p_1 = p_2 = p_3 = \frac{p}{3}$ and $p_4 = 1 - p$	$\left(\frac{1}{3}\right)^{S-z_4} \frac{(S-z_4)!}{(S+1)z_1!z_2!z_3!}$	`-(S-z[4])*log(3)+lfactorial(S-z[4])-log(S+1)-sum(lfactorial(z[1:3]))`
$M_6: p_1 = pp_a, p_2 = p_3 = \frac{p(1-p_a)}{2}$ and $p_4 = 1 - p$	$\left(\frac{1}{2}\right)^{z_2+z_3} \frac{(z_2+z_3)!}{(S+1)(S-z_4+1)z_2!z_3!}$	`-(z[2]+z[3])*log(2)+lfactorial(z[2]+z[3])-log(S+1)-log(S-z[4]+1)-sum(lfactorial(z[c(2,3)]))`
$M_7: p_2 = pp_a, p_1 = p_3 = \frac{p(1-p_a)}{2}$ and $p_4 = 1 - p$	$\left(\frac{1}{2}\right)^{z_1+z_3} \frac{(z_1+z_3)!}{(S+1)(S-z_4+1)z_1!z_3!}$	`-(z[1]+z[3])*log(2)+lfactorial(z[1]+z[3])-log(S+1)-log(S-z[4]+1)-sum(lfactorial(z[c(1,3)]))`
$M_8: p_3 = pp_a, p_1 = p_2 = \frac{p(1-p_a)}{2}$ and $p_4 = 1 - p$	$\left(\frac{1}{2}\right)^{z_1+z_2} \frac{(z_1+z_2)!}{(S+1)(S-z_4+1)z_1!z_2!}$	`-(z[1]+z[2])*log(2)+lfactorial(z[1]+z[2])-log(S+1)-log(S-z[4]+1)-sum(lfactorial(z[c(1,2)]))`
$M_9: p_1 = pp_a, p_2 = pp_b, p_3 = p(1-p_a-p_b)$ and $p_4 = 1 - p$	$\frac{2}{(S+1)(S-z_4+2)(S-z_4+1)}$	`log(2)-log(S+1)-log(S-z[4]+2)-log(S-z[4]+1)`
