# Supplementary material for: A Bayesian Approach to Analyse Genetic Variation within RNA Viral Populations
Source: PLoS Comput Biol. 2011 Mar 31;7(3):e1002027. doi: 10.1371/journal.pcbi.1002027 (PMC3068928; doi:10.1371/journal.pcbi.1002027)
Supplement: Protocol S1 — Derivations of marginal distributions of the data and a protocol for dealing with multiple viral samples. (PDF) [file pcbi.1002027.s002.pdf]

Supplementary information: A Bayesian approach to analyse genetic variation within RNA viral populations.

Trevelyan J. McKinley, Pablo R. Murcia, Julia R. Gog,
Mariana Varela and James L. N. Wood

Derivations of marginal distributions of data

For a given model M_k, we can use Bayes' Theorem to define the marginal distribution of the data under the given model as:

$$P\left(D|M_k\right) = \int_\Theta P\left(D|\theta_k, M_k\right)\pi\left(\theta_k|M_k\right)d\theta_k$$

where θ_k represents the parameters of model k on parameter space Θ, $\pi\left(\theta_k|M_k\right)$ is the prior distribution for the parameters given M_k, and $P\left(D|\theta_k, M_k\right)$ is the likelihood of the data given the parameters under model k. So this marginal distribution averages over the whole of the unknown parameter space.

Table S1 gives $P\left(D|M_k\right)$ for each of the models discussed in the Materials and Methods, assuming data $D = \{S, z_1, z_2, z_3, z_4\}$ for a single viral sample. Below we discuss a few examples to illustrate how these functions are derived.

In order to fully specify these models it is necessary to choose prior distributions for the unknown parameters θ_k, which in this case are all proportions. For M_0 there are no unknown parameters, and so no priors are necessary. For all other models we use uninformative Dirichlet distributions in which the proportions are uniform but constrained to sum to unity. Therefore in general, the probability density function for variables $p_0, \ldots, p_k$, with parameters $\alpha_0, \ldots, \alpha_k$ is:

$$P\left(p_0, \ldots, p_k; \alpha_0, \ldots, \alpha_k\right) = \frac{1}{B\left(\alpha\right)}\prod_{j=1}^{k} p_j^{\alpha_j - 1},$$

where $\sum_{j=1}^{k} p_j = 1$ and

$$B\left(\alpha\right) = \frac{\prod_{j=1}^{k}\Gamma\left(\alpha_j\right)}{\Gamma\left(\sum_{j=1}^{k}\alpha_j\right)},$$

such that $\Gamma(k) = (k-1)!$. In each case here we choose $\alpha_0 = \ldots = \alpha_k = 1$, and so the prior simplifies to $P\left(p_0, \ldots, p_k; \alpha_0, \ldots, \alpha_k\right) = (k-1)!$. It is worth noting at this stage that the p_js can be given unequal weight through this form of prior if, for example, there is *a priori* information regarding mechanisms such as codon bias.

Hence for M_0 there are no free parameters, and so

$$P\left(D|M_0\right) = P\left(D|p^*, M_0\right) = \frac{S!}{z_1!z_2!z_3!z_4!}\left(\frac{p^*}{3}\right)^{S-z_4}\left(1-p^*\right)^{z_4}.$$

For M_1 we constrain the proportion of mutations at a site to equal the overall mutation rate p^*, but assume that $p_1 \neq p_2 = p_3$. A sensible way to model this is to introduce a dummy parameter p_a that

models the proportion of the p^* mutations that are of type 1, and therefore $1-p_a$ corresponds to the proportion of the p^* mutations that are of types 2 or 3. The model is therefore specified as $p_1 = p^* p_a$, $p_2 = p_3 = \frac{p^*(1-p_a)}{2}$ and $p_4 = 1 - p^*$, such that $\sum_{j=1}^{4} p_j = 1$. Hence

$$\begin{aligned} P(D|M_1) &= \int_0^1 P(D|p^*, p_a, M_1)\,\pi(p_a, 1-p_a|M_1)\,dp_a, \\ &= \left(\frac{1}{2}\right)^{z_2+z_3} \frac{S!}{z_1!z_2!z_3!z_4!} (p^*)^{S-z_4} (1-p^*)^{z_4} \int_0^1 p_a^{z_1} (1-p_a)^{z_2+z_3}\,dp_a, \end{aligned}$$

since $\pi(p_a, 1-p_a|M_1) = \text{Dirichlet}(1,1) = 1$. The integral is just a beta function, with non-negative integer coefficients, so may be written in terms of factorials to give

$$\begin{aligned} P(D|M_1) &= \left(\frac{1}{2}\right)^{z_2+z_3} \frac{S!}{z_1!z_2!z_3!z_4!} (p^*)^{S-z_4} (1-p^*)^{z_4} \frac{z_1!\,(z_2+z_3)!}{(S-z_4+1)!}, \\ &= \left(\frac{1}{2}\right)^{z_2+z_3} \frac{S!\,(z_2+z_3)\,!}{z_2!z_3!z_4!\,(S-z_4+1)!} (p^*)^{S-z_4} (1-p^*)^{z_4}. \end{aligned}$$

Similar results hold for models M_2 and M_3. Model M_4 has the same constraint with regard to the total mutation rate but allows each of $p_0, \ldots, p_3$ to differ. The model is therefore specified as $p_1 = p^* p_a$, $p_2 = p^* p_b$, $p_3 = p^*(1-p_a-p_b)$ and $p_4 = 1-p^*$. Hence $\sum_{j=1}^{4} p_j = 1$ and

$$\begin{aligned} P(D|M_4) &= \int_0^1 \int_0^{1-p_a} P(D|p^*, p_a, p_b, M_4)\,\pi(p_a, p_b, 1-p_a-p_b|M_4)\,dp_b dp_a, \\ &= 2\frac{S!}{z_1!z_2!z_3!z_4!} (p^*)^{S-z_4} (1-p^*)^{z_4} \int_0^1 p_a^{z_1} \int_0^{1-p_a} p_b^{z_2} (1-p_a-p_b)^{z_3}\,dp_b dp_a, \end{aligned}$$

since $\pi(p_a, p_b, 1-p_a-p_b|M_4) = \text{Dirichlet}(1,1,1) = 2$. This can be solved in the same way as before to give

$$P(D|M_4) = 2\frac{S!}{z_4!\,(S-z_4+2)!} (p^*)^{S-z_4} (1-p^*)^{z_4}.$$

In order to calculate $P(D|M_k)$ for $M_5, \ldots, M_9$ we use the same framework but this time constrain the total mutation rate to sum to a value p rather than p^*. Hence we introduce an additional variable to which we assign a Uniform(0,1) prior probability [equivalent to Dirichlet(1,1)]. For brevity we show the calculation for M_9 only, which is specified as $p_1 = pp_a$, $p_2 = pp_b$, $p_3 = p(1-p_a-p_b)$ and $p_4 = 1-p$. Hence $\sum_{j=1}^{4} p_j = 1$ and

$$\begin{aligned} P(D|M_9) &= \int_0^1 \int_0^1 \int_0^{1-p_a} P(D|p, p_a, p_b, M_9)\,\pi(p, 1-p|M_9)\,\pi(p_a, p_b, 1-p_a-p_b|M_9)\,dp_b dp_a dp, \\ &= 2\frac{S!}{z_1!z_2!z_3!z_4!} \int_0^1 p^{S-z_4} (1-p)^{z_4} \int_0^1 p_a^{z_1} \int_0^{1-p_a} p_b^{z_2} (1-p_a-p_b)^{z_3}\,dp_b dp_a dp, \\ &= \frac{2}{(S-z_4+2)\,(S-z_4+1)\,(S+1)}. \end{aligned}$$

The other functions in Table S1 follow similarly. These functions are straightforward to calculate in R, though to protect against numerical overflow it is sensible to do this initially on the log-scale using the `lgamma()` or `lfactorial()` functions (see Table S1).

Dealing with multiple samples

As an illustration of these calculations for multiple sets of sequence samples, consider the two-sample case. The data for each sample are denoted $D_1 = \{S_1, z_{11}, z_{12}, z_{13}, z_{14}\}$ and $D_2 = \{S_2, z_{21}, z_{22}, z_{23}, z_{24}\}$ respectively. In any case where the two samples are assumed to come from independent populations,

e.g. models $M_{i,j}$ where $i \neq j$, or $M_{ia,ib}$, then the required probability is just the product of the contributions from the individual samples. So $P(D_1, D_2|M_{i,j}) = P(D_1|M_i)\,P(D_2|M_j)$ when $i \neq j$, or $P(D_1, D_2|M_{ia,ib}) = P(D_1|M_i)\,P(D_2|M_i)$ when $i = j$ but the populations are different (e.g. Figure 2B). In these cases we integrate over two distinct populations.

In the case where the samples are considered to be independent draws from the same population, then we integrate over a single population. To illustrate these difference consider models $M_{5a,5b}$ and $M_{5a,5a}$ (Figure 2). These assume the same background population structure, but in the first case we assume the background population parameters are different and in the second we assume they are the same. Hence

$$\begin{aligned}
P(D_1, D_2|M_{5a,5b}) &= \int_0^1 \int_0^1 P(D_1, D_2|p_1, p_2, M_5)\,\pi(p_1, p_2|M_5)\,dp_2 dp_1, \\
&= \int_0^1 P(D_1|p_1, M_5)\,\pi(p_1, 1-p_1|M_5)\,dp_1, \\
&\qquad \times \int_0^1 P(D_2|p_2, M_5)\,\pi(p_2, 1-p_2|M_5)\,dp_2, \\
&= P(D_1|M_5)\,P(D_2|M_5)\,.
\end{aligned}$$

Whereas

$$\begin{aligned}
P(D_1, D_2|M_{5a,5a}) &= \int_0^1 P(D_1, D_2|p, M_5)\,\pi(p, 1-p|M_5)\,dp, \\
&= \int_0^1 P(D_1|p, M_5)\,P(D_2|p, M_5)\,\pi(p, 1-p|M_5)\,dp.
\end{aligned}$$

This latter model can be written

$$\begin{aligned}
&P(D_1, D_2|M_{5a,5a}) \\
&\quad = \frac{S_1!}{z_{11}!z_{12}!z_{13}!z_{14}!}\frac{S_2!}{z_{21}!z_{22}!z_{23}!z_{24}!}\int_0^1 \left(\frac{p}{3}\right)^{z_{11}+z_{21}+z_{12}+z_{22}+z_{13}+z_{23}} (1-p)^{z_{14}+z_{24}}\,dp, \\
&\quad = \frac{S_1!}{z_{11}!z_{12}!z_{13}!z_{14}!}\frac{S_2!}{z_{21}!z_{22}!z_{23}!z_{24}!}\frac{(z_{11}+z_{21})!\,(z_{12}+z_{22})!\,(z_{13}+z_{23})!\,(z_{14}+z_{24})!}{(S_1+S_2)!}P(D_1+D_2|M_5)\,, \\
&\quad = \frac{\prod_{j=1}^4 \binom{z_{1j}+z_{2j}}{z_{1j}}}{\binom{S_1+S_2}{S_1}}P(D_1+D_2|M_5)\,,
\end{aligned}$$

where $D_1 + D_2 = (S_1 + S_2, z_{11} + z_{21}, z_{12} + z_{22}, z_{13} + z_{23}, z_{14} + z_{24})$. The same structure holds for all models M_k such that $k > 0$.
